# Ultra-high frequency repetitive TMS at subthreshold intensity induces suprathreshold motor response via temporal summation

**DOI:** 10.1088/1741-2552/ad692f

**Published:** 2024-08-08

**Authors:** Hieu Nguyen, Charlotte Qiong Li, Samantha Hoffman, Zhi-De Deng, Yihong Yang, Hanbing Lu

**Affiliations:** 1 Magnetic Resonance Imaging and Spectroscopy Section, Neuroimaging Research Branch, National Institute on Drug Abuse, Intramural Research Program, National Institutes of Health, Baltimore, MD, United States of America; 2 Computational Neurostimulation Research Program, Noninvasive Neuromodulation Unit, Experimental Therapeutics and Pathophysiology Branch, National Institute of Mental Health, National Institutes of Health, Bethesda, MD, United States of America

**Keywords:** rTMS, motor evoked potential, MEP, action potential, membrane time constant

## Abstract

*Objective.* The transcranial magnetic stimulation (TMS) coil induces an electric field that diminishes rapidly upon entering the brain. This presents a challenge in achieving focal stimulation of a deep brain structure. Neuronal elements, including axons, dendrites, and cell bodies, exhibit specific time constants. When exposed to repetitive TMS pulses at a high frequency, there is a cumulative effect on neuronal membrane potentials, resulting in temporal summation. This study aims to determine whether TMS pulse train at high-frequency and subthreshold intensity could induce a suprathreshold response. *Approach.* As a proof of concept, we developed a TMS machine in-house that could consistently output pulses up to 250 Hz, and performed experiments on 22 awake rats to test whether temporal summation was detectable under pulse trains at 100, 166, or 250 Hz. *Main results.* Results revealed that TMS pulses at 55% maximum stimulator output (MSO, peak d*I*/d*t* = 68.5 A/*μ*s at 100% MSO, pulse width = 48 *μ*s) did not induce motor responses with either single pulses or pulse trains. Similarly, a single TMS pulse at 65% MSO failed to evoke a motor response in rats; however, a train of TMS pulses at frequencies of 166 and 250 Hz, but not at 100 Hz, successfully triggered motor responses and MEP signals, suggesting a temporal summation effect dependent on both pulse intensities and pulse train frequencies. *Significance.* We propose that the temporal summation effect can be leveraged to design the next-generation focal TMS system: by sequentially driving multiple coils at high-frequency and subthreshold intensity, areas with the most significant overlapping E-fields undergo maximal temporal summation effects, resulting in a suprathreshold response.

## 1. Introduction

Transcranial magnetic stimulation (TMS) operates by delivering a brief yet strong electric current to a coil, which, through the process of electromagnetic induction, generates an electric field (E-field) in the brain. Theoretically, it has been established that the induced E-field is consistently more powerful on the surface of the brain, diminishing progressively as it extends from superficial to deeper regions of the brain [1]. As a result, key brain regions known to be critically involved in major depression and drug use disorders, such as the subgenual anterior cingulate cortex and the ventral striatum [2, 3], are more than 5 cm from the brain surface and not directly accessible using existing TMS technologies. Compared to conventional figure-of-eight coils, commercial H coils can access deeper brain regions [4]. However, H coils also stimulate a significantly greater volume of superficial brain tissue, as suggested previously [5].

From a biophysical point of view, neuronal elements, such as axons, dendrites, and cell bodies, may be modeled as lumped resistor-capacitor circuits [6], each having specific time constants (*τ*) [7]. Previous studies mapped *τ* ranging from 10 *µ*s to 60 ms [8–10]. On a macroscopic scale, the effects of successive pulses on neuronal membrane potentials can be mathematically modeled as [11]:

\begin{align*}{\text{ }}\Delta V &amp; = p\left( t \right) \otimes h\left( t \right).\end{align*}

Here $ \otimes $ stands for convolution; $p\;(t)$ represents TMS pulse train; $h\left( t \right){\text{ }}$ is the impulse response function. To first-order, $h\left( t \right){\text{ }}$ can be approximated by an exponential decay function: $h\left( t \right) = A*{e^{ - \frac{t}{\tau }}}$, where $A$ is a constant specific to neuronal properties. It can be readily proven that when TMS pulses are delivered repetitively at a high frequency and when *τ* reaches a sufficient duration, successive TMS pulses produce a cumulative effect on $\Delta V$ that manifests as temporal summation.

The concept of temporal summation was demonstrated with transcranial electric stimulation (TES) in rats and in humans by Vöröslakos *et al* [10]: a train of short electric pulses was applied, individual pulse alone was unable to induce suprathreshold response due to the low current intensity applied; successive pulses produced an accumulative effect that eventually led to neuronal firing. Furthermore, Vöröslakos *et al* applied TES pulses through 3 alternating electrode pairs (400 kHz for each pair), producing E-fields that overlapped deep in the rat brain; neurons in the overlapped brain regions experienced all 3 E-fields generated by the 3 alternating electrode pairs, and were more likely to reach threshold potential. Thus, Vöröslakos *et al* were able to produce suprathreshold electric stimulation in a focused region deep in the brain through temporal summation.

**Figure 1.**
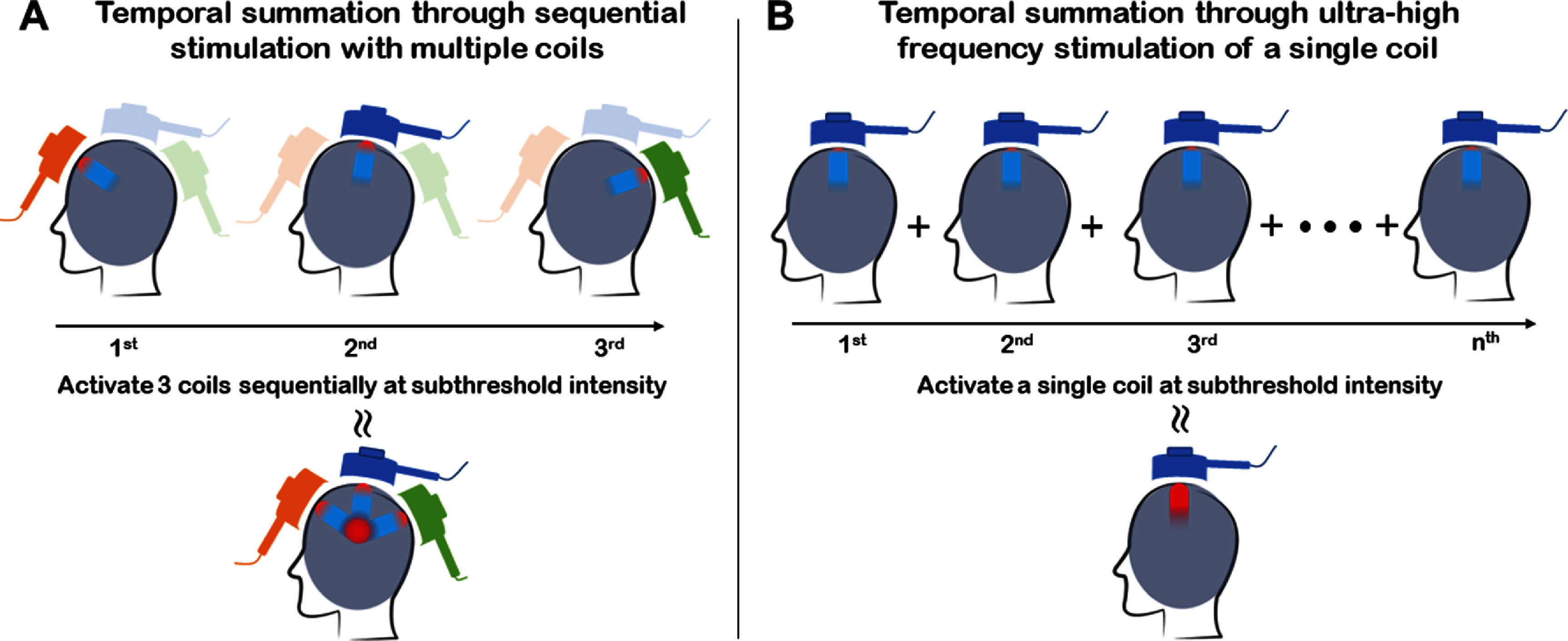
Conceptual illustration of temporal summation for focal and deep TMS. (A) Three distinct TMS coils, each generating its electric field within the brain, are activated in sequence by three separate stimulators at subthreshold levels. When the intervals between consecutive pulses delivered by two separate coils are adequately brief, areas with the highest electric field overlap experience maximal temporal summation effects, resulting in a suprathreshold response. (B) An experiment designed to prove the concept of temporal summation effect in TMS: a train of pulses is delivered to one single coil at subthreshold intensity and high frequency. A suprathreshold response caused by a subthreshold pulse train is the result of temporal summation.

In principle, the temporal summation effect is not limited to TES; TMS should be able to produce a similar effect under the right conditions: successive TMS pulses are applied to the brain through multiple TMS coils, each coil producing its E-field, neurons within the region of maximum E-field overlap experience maximum temporal summation effect, and thus could potentially lead to suprathreshold response. This effect is conceptually illustrated in figure 1(A): 3 TMS coils are driven by 3 separate stimulator units that turn ON and OFF at specified time intervals as needed; each coil produces a distinct E-field, and the red area indicates maximal overlap among the 3 E-fields. Temporal summation in electrical stimulation of peripheral nerve fibers was initially explored in a theoretical study by Reilly [12] and disclosed in a patent application within the framework of TMS [13].

However, there are two important questions that need to be demonstrated experimentally before one can achieve focused deep TMS through temporal summation: (1) What is the minimum TMS frequency necessary to produce a temporal summation effect strong enough for suprathreshold response? (2) How to design TMS coils that produce an overlapped E-field of sufficient intensity and small volume?

As a first step, this study aims to address the following question: Is it feasible to detect the temporal summation effect of TMS pulses *in vivo*? As a proof of concept, we propose to apply TMS pulses at low intensity and ultra-high frequency (*f*) (in contrast to conventional high-frequency rTMS, which usually operates at frequencies below 50 Hz). These pulses are applied to the same coil (see figure 1(B) for illustration). Neurons within the E-field induced by the same TMS coil will experience all pulses and produce a temporal summation effect if it exists and is detectable. To achieve this objective, we developed a TMS machine in-house which outputs consistent pulses up to 250 Hz, and performed *in-vivo* experiments in awake rats using this machine; results demonstrate that suprathreshold response can be elicited by a train of pulses at ultra-high frequencies and subthreshold intensities, leading to motor responses that could be observed visually and quantified through motor-evoked potential (MEP) recordings.

Numerous endeavors have been made to model neuronal reactions to external electromagnetic fields [7, 12, 14, 15]. Recent advances have integrated realistic neuronal morphology and biophysical characteristics [16–20]. We conducted simulations to explore the theoretical potentials of temporal summation effects using a realistic neuron model [18]; we parametrically varied pulse frequencies and E-field strengths; results reveal distinct temporal summation effects. Our study opens the potential to design the next-generation TMS machine to produce focused stimulation deep in the brain based on the principle of temporal summation.

## 2. Materials and methods

### 2.1. Development of ultra-high frequency TMS stimulator

We developed an in-house stimulator that outputs TMS pulses at ultra-high frequency. The circuit topography was similar to our previous report [21]. The high-voltage switching circuit was based on insulated gate bipolar transistor (IGBT) technology [22]. Major changes were: 1) we reduced the energy-storing capacitors C1 and C2 from 190 *µ*F to 30 *µ*F (model UP2_BN306J, 1200VDC, Electronic Concepts, Inc., New Jersey, USA); (2) We employed more powerful power supply units (PSUs): positive power supply unit delivers a peak capacitor charging power of 9000 J/s, and a maximum charging current of 3.6 A (model 802L-5kV-POS, TDK-Lambda Americas, New Jersey, USA); negative power supply delivers a peak charging power of 1650 J/s, and maximum charging current of 1.1 A (model 152A-3kV-NEG, TDK-Lambda Americas, New Jersey, USA). (3) We replaced the rat-specific TMS coil with a human TMS coil (inductance *L* = 16 *µ*H, D70 Alpha Coil, The Magstim Company Ltd, Whitland, UK); (4) the snubber circuit was adjusted accordingly to suppress circuit spiking during IGBT switching.

The TMS pulse paradigms were programmed based on a microprocessor (Atmel ATmega328), which controlled PSU output voltage, capacitor charging and discharging, and the timing of IGBT ON/OFF (model FZ1200R45KL3_B5, Infineon Transistors, IH Series). The low-voltage control unit was electrically isolated from the high-voltage unit through optic coupling.

**Figure 2.**
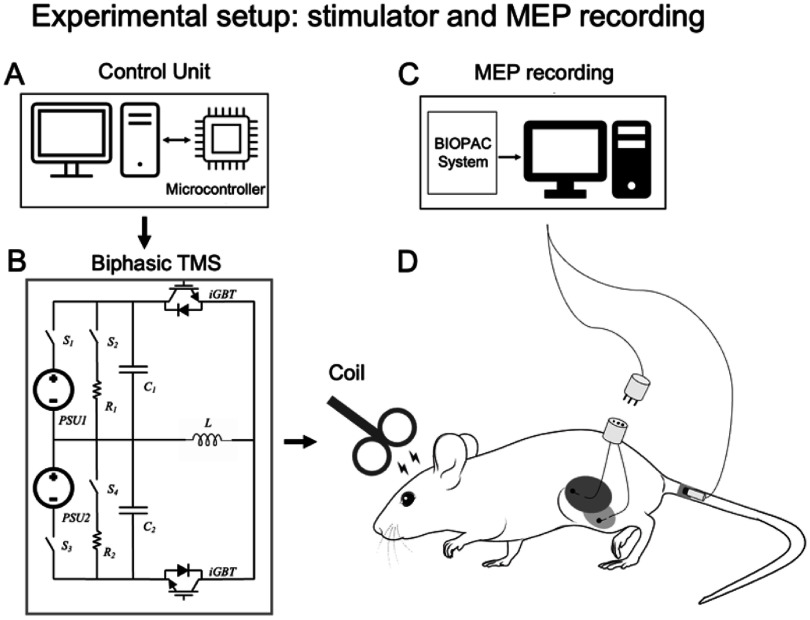
Illustration of experimental setup. TMS control signals and pulse paradigms were generated with a microcontroller unit (A)–(B). TMS pulses were delivered to the rat brain via a Magstim Alpha D70 coil. Microwire electrodes were implanted on rat leg muscles to record electromyographic signal using the BIOPAC recording system (C)–(D).

A Rogowski current waveform transducer (model CWT30, peak current 6000 A, Power Electronic Measurements Ltd, Nottingham, UK) was used to measure the coil current output. This stimulator can deliver stable TMS pulses up to 250 Hz with a maximal current of 1500 A. The LC oscillatory circuit had an intrinsic period of 137.6 *μ*s. IGBT semiconductor switches were turned ON and OFF to produce a pulse duration of 48 *μ*s, resulting in a near-rectangular biphasic E-field instead of a damped cosine E-field waveform [21, 22]. The maximum d*I*/d*t* was 68.5 A/*μ*s at maximum machine output (MSO). Figures 2(A) and (B) illustrate the circuit topography of the ultra-high frequency TMS stimulator.

### 2.2. Animal experiments

Human studies recorded motor evoked potential (MEP) signal from effector muscle to assess TMS effects in the motor cortex [23]. We adopted a similar approach and recorded MEP signal in awake rats to quantitatively assess TMS-induced motor response. Microelectrodes were constructed in-house and were longitudinally implanted in the rats. This method allowed for repeated MEP signal recording across animals and TMS sessions for up to 1 month. Procedures for electrode construction, surgical implantation, and TMS administration were detailed previously [21, 24]. All procedures were approved by the Animal Care and Use Committee at the National Institute on Drug Abuse, NIH.

#### 2.2.1. Electrode implantation for MEP recording and TMS administration

Using aseptic procedures and under isoflurane anesthesia, microelectrodes were surgically implanted into the biceps femoris and gastrocnemius muscles of the rat hindlimb. The other ends of the electrodes were interfaced to an electrode pedestal (MS363, P1 Technologies, USA), which was anchored on the rat’s lower back as a ‘backmount’. To record the MEP signals, a male connector (part #: 363-441/6, P1 Technologies, USA) was attached to the pedestal to interface the electrodes to a BIOPAC system (BIOPAC Systems Inc., CA, USA). An EEG pad adhered to a shaved portion of the rat’s tail to serve as the ground electrode. The MEP signal was band-pass filtered (100–5000 Hz), amplified by 2000, and sampled at 10 kHz. Experiments started after one week of surgical recovery. Figures 2(C) and (D) illustrate the MEP recording setup. Awake rats do not readily comply with the motionless requirement for TMS administration. We habituated rats to handling and the TMS environment, including acoustic noise, for 7 days using the procedures reported previously [25].

#### 2.2.2. Experimental design

Given the fact that our in-house developed TMS machine outputs stable pulses up to 250 Hz, we applied the following 4 pulse paradigms: (i) single pulse (SP); (ii) pulse trains, each consisting of 10 pulses, inter-pulse interval (IPI) = 10, 6 or 4 ms, corresponding to the pulse frequency of 100, 166 and 250 Hz, respectively (noted as TS100, TS166, and TS250 for convenience). We employed a within-subject design: rats received single-pulse TMS on the first day to determine motor threshold. The power level that induced motor response in 5 out of 10 pulses was considered motor threshold; there was excellent correspondence between visually observed motor responses and MEP signal, as reported previously [21, 25]. High-frequency TMS pulses (TS100, TS166, or TS250) were applied to the rats in the 3 days that followed, with one pulse type on a given day. The orders of the 3 high-frequency pulse types were randomized among the rats.

**Figure 3.**
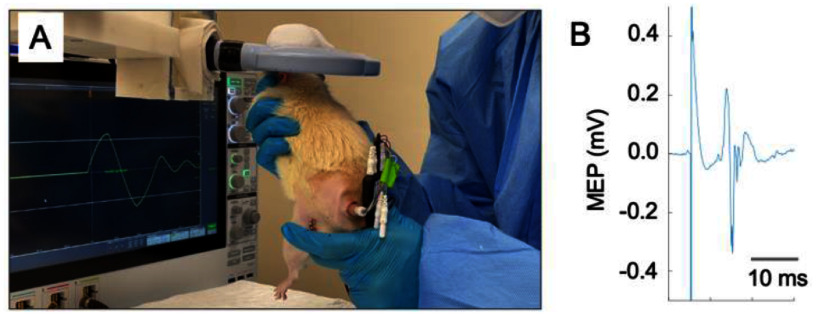
(A) Illustration of a rat receiving TMS. Electrodes were implanted on the left hindlimb and back-mounted. An oscilloscope displayed coil current. Dry ice on top of the coil prevented overheating resulting from the high-frequency pulse train. (B) Shows a typical motor-evoked potential (MEP) signal induced by a single-pulse TMS at 90% maximum stimulator output.

Our stimulator required a low inductance coil to deliver high-frequency TMS pulses. The rat-specific TMS coil we had previously developed had a high inductance (*L* = 47 *µ*H) and a relatively high ohmic loss [26]. Consequently, it was impractical to deliver stable TMS pulses at 166 Hz or higher using the stimulator described above. We thus opted to utilize a commercial figure-of-eight coil designed for humans (Model: D70 Alpha, Magstim Company Ltd, UK). The center of this coil was marked. The caudal boundaries of rat eyes were used as the anatomical reference, which approximately corresponded to −5 mm from bregma [27]. The hindlimb representation of the motor cortex is about 7 mm caudal to this reference (+2 mm from bregma [28]). The rats were manually held with their hindlimb motor cortex region positioned under the center of the coil. Their ears were manually blocked to minimize the effects of the acoustic noises from the TMS coil. Consequently, the rats did not exhibit the startle responses that would otherwise often be triggered if their ears were not blocked. Figure 3 shows a rat undergoing TMS stimulation. Due to the relatively large size of the coil compared to a rat’s brain, it is expected that a significant portion of the rat’s brain received stimulation [29]. Dry ice was placed on top of the coil to prevent overheating from high-frequency pulse trains.

Adult male Sprague-Dawley rats weighing 350–450 grams were used in this study (*n* = 22 in total). Since TS250 was most likely to induce a temporal summation effect, initial experiments focused on a 250 Hz pulse train: 14 rats received TMS pulses at 3 power levels: 75%, 90%, and 100% MSO. For convenience, animals assigned in this study were considered cohort 1.

**Figure 4.**
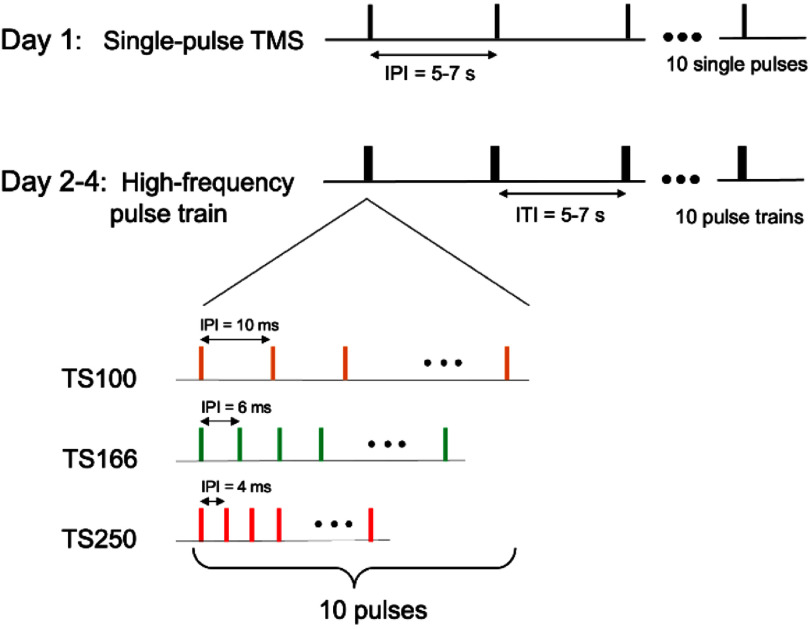
Experimental design. Single-pulse TMS was applied to map motor threshold on day 1. Rats underwent sessions of high-frequency pulse trains on days 2-4, one type of pulse train (TS100, TS166, or TS250) on a given day, randomized across animals. There were 7 power levels at each frequency; ten pulse trains were administered at each power level, and each pulse train consisted of ten pulses. The order of the 7 power levels was randomized as well. There were 5 min intervals between these power levels to minimize potential aftereffects from prior pulse trains. Abbreviations: TS100, TS166, and TS250 denote pulse train at 100, 166, and 250 Hz, respectively. Abbreviations: IPI, inter-pulse interval. ITI, inter-train interval.

To further explore the TMS power and frequency-dependence of the temporal summation effect, another cohort of rats (cohort 2, *n* = 8) received TMS pulses at 3 frequencies (TS100, TS166, TS250), one type of pulse train on a given day, each was delivered at 7 power levels (55%, 65%, 75%, 85%, 90%, 95%, and 100% MSO). At each power level, a total of 10 pulses (single-pulse TMS) or 10 pulse trains (TS100, TS166, and TS250) were delivered with an inter-train interval (ITI) of 5–7 s. The order of TMS power levels was randomized, with 5 min intervals between power levels to minimize potential aftereffects from prior pulse trains. Additionally, pulse train types were randomized across animals to minimize potential cumulative TMS effects from previous days when comparing temporal summation across pulse trains. Figure 4 illustrates experimental timelines. For each animal, it took about 15 min to complete the single-pulse TMS experiments (on day 1), and 45 min to complete the high-frequency pulse train experiments (on days 2-4), with a total of 2 weeks (including surgical preparation) to complete the experiments.

TMS pulse intensity at 55% MSO had a peak d*I*/d*t* of 37.7 A/*μ*s; no motor response or MEP signal was detected in any of the rats under either single-pulse or pulse train conditions (see results). Data acquired under 55% MSO served as a positive control.

#### 2.2.3. Data analysis

We quantified the temporal occurrences of the MEP signals. This was done as follows: first, the MEP signals under each power level and each pulse train type were identified. For each animal, the MEP signal at motor threshold was identified based on single-pulse TMS data acquired on Day 1 (see figure 4). In the absence of an MEP signal, high-frequency pulse trains induced regular artefactual patterns in the recording channel (see Results below). Deviation from the regular patterns, with a peak-to-peak amplitude greater than the MEP signal at motor threshold, was considered the occurrence of MEP induced by high-frequency pulse trains.

We next calculated the frequency of MEP occurrences for each power level and each pulse train type as follows: Each pulse train consisted of 10 pulses, and the frequency of MEP occurrence following the *i*th pulse was calculated using the formula: $\frac{{{N_i}}}{{{M_{{\text{total}}}}}} \times 100\% $. Here, ${N_i}$ represents the total number of MEP occurrences following the *i*th pulse, counted across animals: ${N_i} = \sum\nolimits_{n = 1}^8 {{O_{n,i}}} $ (8 rats, *i* = 1, 2, …, 10), where ${O_{n,i}}$ denotes the number of MEP occurrences for the *n*th rat at the *i*th pulse. ${M_{{\text{total}}}}$ is the total number of MEP occurrences across all pulse numbers and animals, given by ${M_{{\text{total}}}} = \sum\nolimits_{i = 1}^{10} {{N_i}} $. Finally, the frequency of MEP occurrences following the *i*th pulses was plotted for each power level and each pulse train type.

We also compared the amplitude of the MEP signal across pulse types and TMS power levels based on the method reported previously [21], and are described as follows: the peak-to-peak values of the MEP signals were calculated for each occurrence. The mean and standard deviation of the MEP signals under each condition (pulse type, power level) were calculated across animals. For each pulse type, there was a plot of MEP amplitude (*y*-axis) vs. TMS pulse intensity (*x*-axis). The area-under-curve (AUC) of MEP amplitude-TMS pulse intensity plots were calculated using Python function numpy.trapz(). The resulting AUC values across animals were subjected to non-parametric repeated measures Friedman test with PULSE TYPE as a factor to assess any significant difference among the TMS pulse types. Post-hoc Wilcoxon analyses were subsequently applied to further evaluate differences in MEP responses among stimulus intensities. *P*< 0.05 was considered statistically significant.

In addition to the above non-parametric tests, we also performed parametric tests using two-factorial repeated measures ANOVA to assess the effects of INTENSITY, PULSE TYPE, and their interaction. To ensure normality, a Box-Cox transformation (*β* = 0.1) was applied to the raw MEP data. Post-hoc pairwise two-sided tests with multiple comparisons corrections (Benjamini-Hochberg) were applied to evaluate the effects of PULSE TYPE and INTENSITY. Detailed methods and results of the parametric tests are presented in supplemental materials.

### 2.3. Theoretical simulation

To further explore whether temporal summation as a basic biophysical mechanism is sufficient to induce neuronal firing through a train of subthreshold electromagnetic pulses, we performed simulations on single neurons using the Neuron Modeling for TMS (NeMo-TMS) software package [18]. NeMo-TMS integrates the NEURON simulation environment (Hines, MI, 1997) and the T2N extension of the TREES Toolbox [30], generates accurate neuron models from morphological reconstructions, coupling them to the external E-field induced by TMS, and simulates the cellular and subcellular responses. Specifically, we used the Jarsky model of the hippocampal CA1 pyramidal cells [17, 31]. This model, based on experimental observations, specifies cell types with different Na^+^ and K^+^ channels featuring different properties and conductance. The rationale for choosing Jarsky pyramidal model was as follows: Jarsky cells were built with specific ion channels that reflected anatomical properties; previous computer modeling that examined synaptic activity from perforant-pathway projections to the apical tuft of CA1 pyramidal neurons matched remarkably well with slice physiological data [17].

Since motor response evoked by a train of subthreshold TMS pulses likely resulted from a complex interaction of neuronal network instead of a direct effect of individual neurons (see Discussion), we present the theoretical simulation results in supplemental materials.

## 3. Results

### 3.1. Single-pulse TMS at sub- and supra-threshold intensities

All 22 rats received single-pulse TMS (*n* = 14 in cohort 1 and *n* = 8 in cohort 2). TMS intensity at 55% and 65% MSO did not induce motor response or MEP signal in any of the rats. At 75% MSO, 16 rats did not show motor response, two of which did not show motor response even at 100% MSO; the remaining 6 six rats showed weak motor response and weak MEP signal (less than 50 *µ*V peak-to-peak). Of the 20 rats that showed motor responses, the average MEP signal at motor threshold, defined as the minimal TMS power to induce motor response in 5 out of 10 trials, was 270.7 ± 241.7 *μ*V.

**Figure 5.**
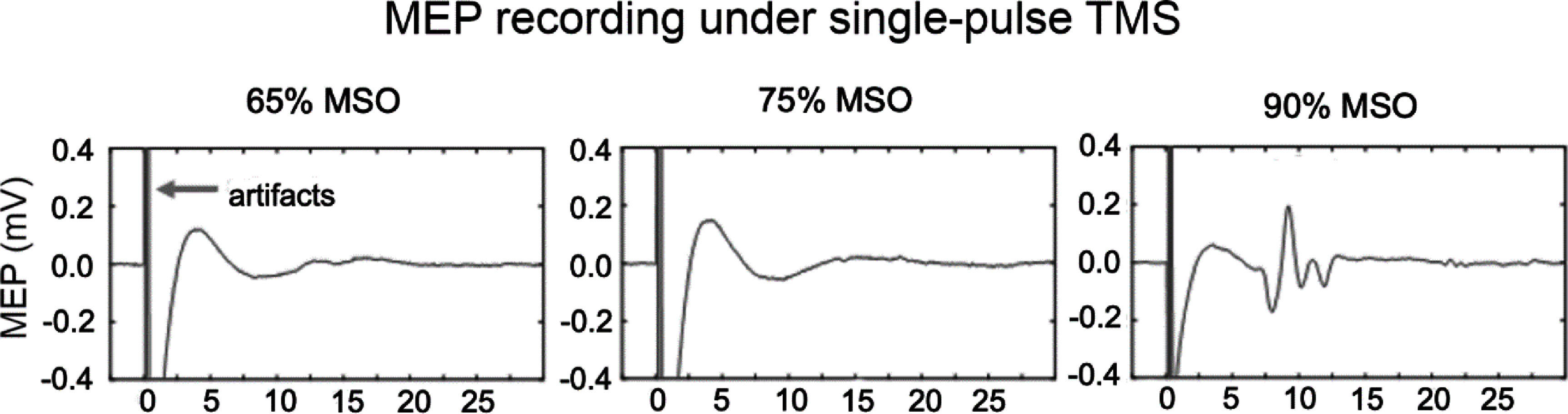
Raw MEP traces from one rat. Single-pulse TMS was applied at 3 stimulation intensities; a distinct MEP signal was detected at 90% maximum stimulator output (MSO) but not at 65% or 75% MSO. The arrow indicates artifacts from the TMS pulse.

Figure 5 shows raw MEP traces from one representative rat. Single-pulse TMS was applied at 65%, 75%, and 90% MSO. Note that distinct MEP appeared only at 90% MSO, manifested as a multi-phasic signal with a latency of about 7 ms, consistent with the previous report [23]. No apparent MEP signal was detected at 65% or 75% MSO. There were artifacts of large amplitude induced by the TMS pulse, indicated by the arrow.

### 3.2. TMS pulse trains at subthreshold intensities induce suprathreshold motor responses

**Figure 6.**
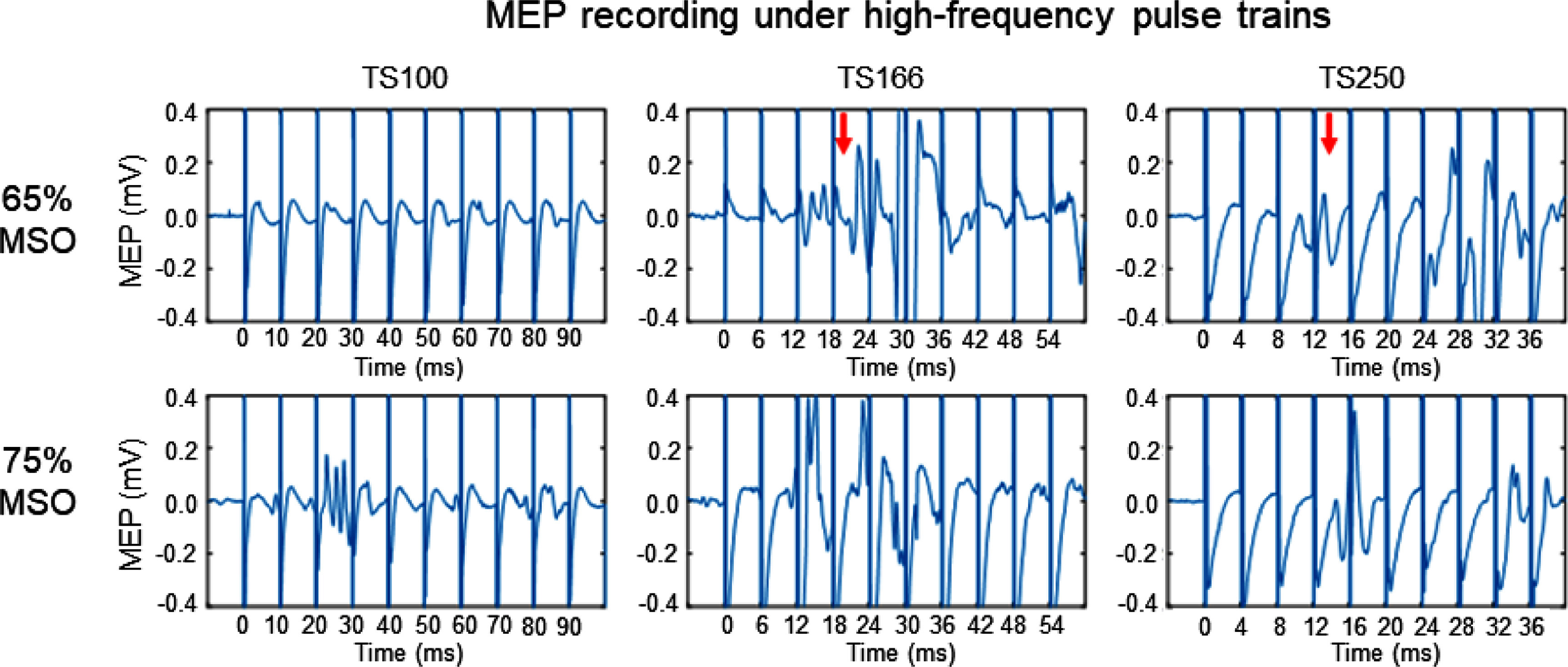
Raw MEP traces under 2 subthreshold intensities (65% and 75% MSO) and 3 high-frequency pulse trains (TS100, TS166, and TS250). Data were recorded from the same rat shown in figure 5. Top panel: at 65% MSO, no MEP signal at TS100, but distinct MEP signals appeared at T166 and TS250 (indicated by the 2 arrows). Bottom panel: at 75% MSO, MEP signals appeared at all 3 frequencies. Abbreviations: TS100, TS166, and TS 250 denote the TMS pulse train at 100, 166, and 250 Hz, respectively.

We next investigated whether a train of TMS pulses at subthreshold intensities could trigger motor response and MEP signal. As an example, figure 6 shows raw EMG recordings from one rat (the same rat in figure 5) using 3 pulse paradigms: TS100, TS166, and TS250. At a TMS intensity of 65%, MEP signal emerged at TS166 and TS250; we also visually observed motor responses, but there was no MEP signal and no motor response at TS100. When pulse intensity increased to 75%, we observed MEP signal and motor responses under all 3 pulse train paradigms.

**Figure 7.**
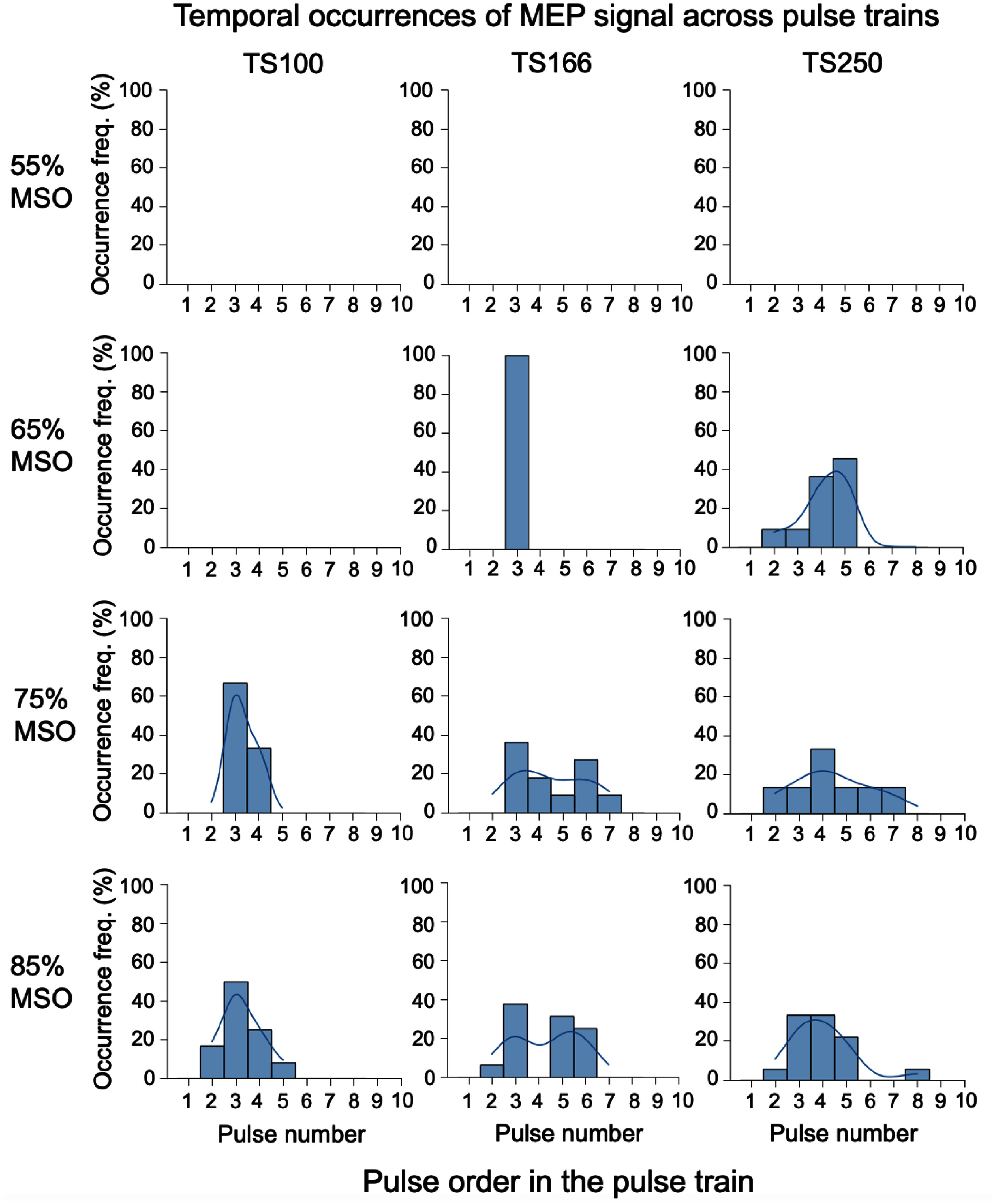
Distribution of the temporal occurrence of the MEP signal across pulse numbers. Each high-frequency pulse train consisted of 10 pulses. Data were collected from animals in cohort 2 (*n* = 8 rats). Note the absence of an MEP signal at 55% MSO pulse intensity, regardless of pulse train frequencies. Single-pulse TMS at 65% MSO was also subthreshold but induced MEP signals when delivered with pulse trains at 166 and 250 Hz, but not at 100 Hz. Abbreviations: MSO: maximal machine output (peak d*I*/d*t* = 68.5 A/*μ*s at 100% MSO). TS100, TS166, TS250: pulse trains at 100, 166, and 250 Hz, respectively.

Figure 7 summarizes the temporal occurrence of the MEP signal across all animals in cohort 2 (*n* = 8). Since the primary objective of this study was to determine if subthreshold intensity TMS pulses, when administered in high-frequency pulse trains, could elicit a suprathreshold motor response, only pulse intensities ranging from 55% to 85% MSO are shown for visual clarity. At a pulse intensity of 55% MSO, no MEP was induced in any of the pulse train types (TS100, TS166, and TS250). When the pulse intensity increased to 65% MSO, MEP signals were observed at TS166 and TS250 but not at TS100. As the pulse intensities further increased to 75% and 85% MSO, MEP signals were present in all three pulse train types.

**Figure 8.**
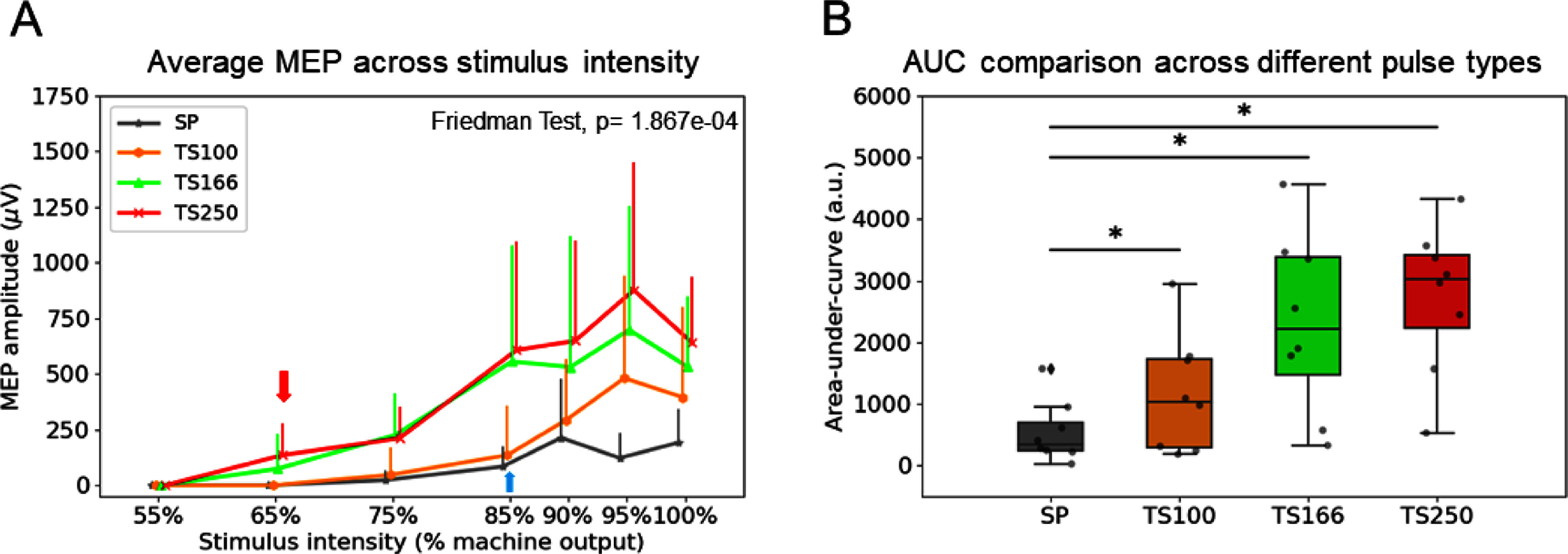
(A) MEP signals at different TMS pulse intensities under 4 different TMS paradigms. Data were averaged from animals in cohort 2 (*n* = 8 rats). (B) Statistical comparison of the AUC values across different pulse types. *, *p* < 0.05, post-hoc Wilcoxon signed-rank tests. Abbreviations: AUC, area-under-curve.

Next, we compared MEP signal amplitudes as a function of TMS pulse intensities; the results are summarized in figure 8(A). Figure 8(B) compares AUC values of MEP amplitudes-pulse intensities among the pulse types using the non-parametric Friedman test with ‘PULSE TYPE’ as a factor. There was a significant main effect (statistic test *Q* = 19.80, *p* = 1.86 × 10^−04^). Post-hoc Wilcoxon signed-rank tests revealed that, compared to single-pulse TMS, AUC values in all 3 pulse train types were significantly higher than in single-pulse: TS100 vs. SP (corrected *p* = 0.046); TS166 vs. SP (corrected *p* = 0.011); TS250 vs. SP (corrected *p* = 0.011) (supplemental table 1).

Two-way repeated measures ANOVA was used to access the significant effects in both ‘PULSE TYPE’ and ‘INTENSITY’ factors. Skewed MEP values were first transformed to normal distribution with a Box-Cox transformation (*β* = 0.1) for parametric statistical analysis (supplemental figure 1). Two-way ANOVA reveals that there were significant main effects in ‘PULSE TYPE’ (*F*-value = 28.861, corrected *p* = 1.55 × 10^−05^) and ‘INTENSITY’ (*F*-value = 46.298, corrected *p* = 8.77 × 10^−08^). Furthermore, there was a significant PULSE TYPE × INTENSITY interaction (*F*-value = 3.296, corrected *p* = 4.72 × 10^−02^) (supplemental tables 2–4).

Based on our theoretical simulations, TS250 was most likely to trigger motor response at subthreshold TMS intensity; we thus applied single-pulse TMS and TS250 on 14 rats (cohort 1), each having 3 pulse intensities (i.e. 75%, 90%, and 100% MSO). Results are summarized in supplemental figure 2, supplemental tables 5 and 6. A pulse intensity of 75% MSO induced weak MEP signal in 4 out of the 14 rats using single-pulse TMS, but TS250 at 75% MSO triggered strong motor responses and MEP signal in all rats. With TS250, pulse trains at 90% and 100% MSO significantly enhanced MEP amplitudes compared to 75% MSO (90% vs. 75% MSO: corrected *p* = 0.012; 100% vs. 75% MSO: corrected *p* = 0.005); there was no difference in MEP signal between pulse trains at 90% and 100% MSO (corrected *p* > 0.67).

## 4. Discussions

In the present study, we developed a TMS machine that delivered stable pulse train up to 250 Hz and performed experiments in awake rats. Experimental results reveal that a single TMS pulse at 65% MSO failed to evoke motor response in rats, as evidenced by the absence of MEP signal. Conversely, a train of TMS pulses at 166 and 250 Hz triggered motor response and MEP signal. We also performed theoretical simulation on hippocampal CA1 Jarsky cell models using the NeMo-TMS package [18]. Hippocampal Jarsky cell models took into consideration of the non-linear properties of the Na^+^ and K^+^ channels and passive leakage of cell membrane, resulting in Hodgkin–Huxley model equations matched remarkably well with experimental data [32]. Our simulations found that a high-frequency pulse train at subthreshold intensity could lead to neuronal firing.

We utilized MEP as the readout signal. Temporal summation, a fundamental biophysical phenomenon, is not confined solely to the motor cortex but also occurs in the cortico-spinal pathway, particularly in the motor neurons of the spinal grey matter. Given that the motor cortex was positioned at the hotspot of the TMS coil, it is reasonable to infer that the action potential originated in the motor cortex, traversed through the corticospinal axons, synapsing with alpha motor neurons of the spinal grey matter, and inducing excitatory postsynaptic potentials on the postsynaptic membrane. Subsequently, this process elicited an action potential in the motor neuron, which propagated to the endplate, ultimately generating an MEP signal. The temporal summation effect at spinal motor neurons was examined theoretically and was supported by experimental data [33]. This phenomenon might hold particular significance when trains of TMS pulses are administered at extremely high frequencies. Special caution is warranted when considering the cortical and spinal origins of the MEP signal in these circumstances.

The data depicted in figure 8 illustrates that TMS at pulse trains of 250 and 166 Hz induces a significantly stronger temporal summation effect compared to 100 Hz, necessitating lower E fields to elicit suprathreshold responses. An open question is whether there is an optimal frequency for temporal summation. A previous theoretical simulation by Reilly reported a temporal summation effect up to 2000 Hz [12]; our simulation on Jarsky hippocampal CA1 pyramidal cell models suggests that temporal summation effects tend to plateau around 500 Hz (supplemental figure 5). Discrepancies between these studies may stem from differing model definitions. Reilly’s theoretical study centered on myelinated peripheral nerve fibers, with the fiber length set to 100 times the fiber diameter. In contrast, our simulation utilized Jarsky’s ‘realistic’ hippocampal CA1 neuron models. Disparities in axon diameter, node count, and notably, the non-linear characteristics and ion channel density, could have contributed to the discrepancies. Nonetheless, both Reilly’s peripheral nerve fiber simulations and our hippocampal CA1 model simulations support temporal summation as a fundamental biophysical property, potentially harnessed to evoke suprathreshold responses through subthreshold, high-frequency pulse trains. Our TMS hardware currently restricts the frequencies to 250 Hz. Future endeavors should aim to extend these frequencies to further explore this phenomenon.

### 4.1. Deep and focal TMS based on temporal summation effect

Experimental demonstration of the temporal summation effect opens a few novel potentials for TMS, one of which is to stimulate a focal brain area that sits deep in the brain. As illustrated in figure 1(A), if multiple TMS coils, each producing its E-field in the brain, are driven sequentially, the brain area that has maximal overlap of the E-fields by the coils will experience the highest temporal summation effects, based on equation (1). Let us assume there are 4 coils, C1, C2, C3, and C4 driven by 4 separate power units sequentially, with an inter-stimulus-interval (ITI) of *T* (frequency *F* = 1/*T*); areas covered by all 4 coils will experience a stimulus frequency of *F*; areas covered by C1 only will experience a stimulus frequency of *F*/4. However, as previously shown mathematically by Heller and Van Hulsteyn [1], the E fields generated by individual coils are stronger in superficial cortex, there are temporal summation effect even at *F*/4. Thus, there are delicate trade-offs between temporal summation effects in the superficial cortex and those in deep brain regions to attain targeted stimulation within the deep brain.

An open question is how to design TMS coils that produce E-field overlap of small volume and high intensity: the small volume of E-field overlap will lead to focused stimulation, and the high intensity is necessary to produce suprathreshold response via temporal summation. TMS coils have been conventionally designed by empirically defining coil current distribution outside the head and iteratively optimizing the current distribution to achieve a designed E-field inside the brain. An alternative approach is to define the desired E-field distribution inside the brain first and then derive the coil current distribution that produces the desired E-field. Several mathematical frameworks have been proposed to address this question [11, 34]. Moreover, achieving temporal summation necessitates the sequential activation of multiple coils at high frequencies (250 Hz or higher). Physically relocating individual coils is impractical under these circumstances; thus, separate stimulators are required to drive them. Notably, the concept of driving two coils sequentially has been integrated into rotational field TMS methods [35]. Recently, advancements in multi-locus TMS technology have introduced stimulators capable of independently operating and driving individual coils, thereby enabling diverse stimulation focal points to be achieved [36].

### 4.2. Previous studies employing high-frequency pulse trains

There have been studies that delivered high-frequency pulse trains; one example is the Quadro-pulse TMS: pulse trains, each consisting of 4 pulses, were delivered every 5 s. This was realized through the sequential activation of 4 TMS power supplies. The inter-pulse intervals ranged from 1.5 ms to 100 ms. Quadro-pulse theta burst was also reported [37]. However, the aim of these studies was to enhance the after-effects of TMS; no temporal summation effect was attempted.

Du *et al* conducted experiments involving two subthreshold intensity pulses and observed a supra-threshold motor response when the inter-pulse-intervals were less than 10 ms, with the most pronounced effects occurring at 1, 1.5, and 3 ms [38]. Furthermore, they reported that the facilitatory response, surpassing the threshold, was noticeable when the pulse intensity exceeded 80% of RMT, while no motor response was detected at intensities of 70% or lower. Our rat data largely corroborates these findings. However, as shown by Vöröslakos *et al* [10] in TES, it is very unlikely to achieve focal and deep TMS using two TMS coils. More recently, Labruna *et al* reported TMS at kilohertz [39]. Nevertheless, the E-field intensity was <10 V/m, making it improbable to elicit a suprathreshold response through temporal summation. Sorkhabi *et al* [40] developed a TMS system that was capable of providing stimulation up to 4 kHz at the max current value of 3600 A. This technology permits future testing of temporal summation effects at higher frequencies.

### 4.3. Safety considerations

The proposed method requires activating multiple TMS coils at ultra-high frequencies and relatively high intensities, potentially elevating the risk of inducing seizures compared to traditional TMS methods. In the study conducted by Labruna *et al*, up to 5 kHz pulses were applied, yet these pulses were of notably low intensity (<10 V/m) [39]. A series of studies by Ugawa and colleagues employing the quadri-pulse paradigm offer insights into the safety aspects of ultra-high frequency TMS [41–43]. In these studies, four pulses at suprathreshold intensities were administered to the human motor cortex, with inter-pulse intervals ranging from 1.5 to 1250 ms. Taking a further stride, Jung *et al* applied quadri-pulse theta bursts to the human motor cortex [37], with each burst comprising four pulses and inter-pulse intervals set at either 1.5 or 5 ms. No severe adverse events were reported in any of these studies. However, it is worth noting that these studies applied high-frequency pulse trains to a single coil. More brain regions will be affected when high-frequency pulse trains are applied to multiple coils. Although the aforementioned studies offered promising safety prospects for TMS at ultra-high frequencies, it is well-established that the risk for seizure induction increases with higher-frequency TMS pulses [44–46]. The proposed method involves applying TMS pulses using multiple coils at ultra-high frequencies, affecting wide brain regions, which may pose a greater risk for seizure induction compared to conventional methods. Therefore, the safety profile of the proposed method must be thoroughly and cautiously evaluated.

### 4.4. Limitations

A human TMS coil was used in this study because of its low inductance, which was necessary for delivering TMS pulse trains at ultra-high frequencies. A large portion of the rat brain was likely stimulated by this coil [29]. The neocortex of the mammalian brain has distinct laminar profiles with different neuronal types forming excitatory-inhibitory local circuits [47, 48]. In addition to cellular elements (dendrites, soma, and axons) of individual cells and anatomical connections among the cells within a region (intra-areal connections), for instance, within the primary motor cortex, there are rich feedforward and feedback axonal projections (inter-areal connections), synapsing with downstream excitatory cells that release the neurotransmitter glutamate and inhibitory interneurons that release the neurotransmitter Gamma-aminobutyric acid (GABA), exciting or inhibiting downstream neurons [49–51]. This cascade of events influences neuronal activity beyond the inherent excitability of individual neurons.

Our theoretical simulation was based on Jarsky CA1 cell model. TMS-induced action potentials consistently initiated from axons, and propagated antidromically. To our knowledge, Jarsky CA1 cell model did not include axonal hillock, a critical structure in initiating actional potential in neurons that propagate orthodromically. While our simulations on Jarsky CA1 cell models reveal clear temporal summation effects, supporting theoretical formation in equation (1), simulation results based on individual neurons cannot be readily extrapolated to explain *in-vivo* suprathreshold neuronal responses evoked by a train of TMS pulses.

## 5. Conclusions

The paper introduces a novel method incorporating ultra-high frequency repetitive TMS. This approach aims to elicit a suprathreshold response by administering a high-frequency TMS pulse train with subthreshold intensity. The hypothesis is supported by *in-vivo* experiments on awake rats and by theoretical simulations on realistic cell models. This approach, if further developed, presents exciting possibilities for advancing future TMS research and developing tools for clinical applications.

## Data Availability

The data cannot be made publicly available upon publication due to legal restrictions preventing unrestricted public distribution. The data that support the findings of this study are available upon reasonable request from the authors.

## References

[1] Heller L, Van Hulsteyn D B (1992). Brain stimulation using electromagnetic sources: theoretical aspects. Biophys. J..

[2] Lu M, Ueno S (2017). Comparison of the induced fields using different coil configurations during deep transcranial magnetic stimulation. PLoS One.

[3] Tzirini M, Roth Y, Harmelech T, Zibman S, Pell G S, Kimiskidis V K, Tendler A, Zangen A, Samaras T (2022). Detailed measurements and simulations of electric field distribution of two TMS coils cleared for obsessive compulsive disorder in the brain and in specific regions associated with OCD. PLoS One.

[4] Roth Y, Zangen A, Hallett M (2002). A coil design for transcranial magnetic stimulation of deep brain regions. J. Clin. Neurophysiol..

[5] Deng Z D, Lisanby S H, Peterchev A V (2013). Electric field depth-focality tradeoff in transcranial magnetic stimulation: simulation comparison of 50 coil designs. Brain Stimul..

[6] Nowak L G, Bullier J (1998). Axons, but not cell bodies, are activated by electrical stimulation in cortical gray matter. II. Evidence from selective inactivation of cell bodies and axon initial segments. Exp. Brain Res..

[7] Basser P J, Roth B J (1991). Stimulation of a myelinated nerve axon by electromagnetic induction. Med. Biol. Eng. Comput..

[8] Spruston N, Jaffe D B, Johnston D (1994). Dendritic attenuation of synaptic potentials and currents: the role of passive membrane properties. Trends Neurosci..

[9] Barker A T, Garnham C W, Freeston I L (1991). Magnetic nerve stimulation: the effect of waveform on efficiency, determination of neural membrane time constants and the measurement of stimulator output. Electroencephalogr Clin. Neurophysiol..

[10] Vöröslakos M (2018). Direct effects of transcranial electric stimulation on brain circuits in rats and humans. Nat. Commun..

[11] Alavi S M M, Vila-Rodriguez F, Mahdi A, Goetz S M (2022). A formalism for sequential estimation of neural membrane time constant and input-output curve towards selective and closed-loop transcranial magnetic stimulation. J. Neural Eng..

[12] Reilly J P (1989). Peripheral nerve stimulation by induced electric currents: exposure to time-varying magnetic fields. Med. Biol. Eng. Comput..

[13] Zangen A, Roth Y, Miranda P C, Hazani D, Hallet M (2011). Transcranial magnetic stimulation system and methods. US7976451B2.

[14] Roth B J, Cohen L G, Hallett M, Friauf W, Basser P J (1990). A theoretical calculation of the electric field induced by magnetic stimulation of a peripheral nerve. Muscle Nerve.

[15] McNeal D R (1976). Analysis of a model for excitation of myelinated nerve. IEEE Trans Biomed. Eng..

[16] Aberra A S, Peterchev A V, Grill W M (2018). Biophysically realistic neuron models for simulation of cortical stimulation. J. Neural Eng..

[17] Jarsky T, Roxin A, Kath W L, Spruston N (2005). Conditional dendritic spike propagation following distal synaptic activation of hippocampal CA1 pyramidal neurons. Nat. Neurosci..

[18] Shirinpour S, Hananeia N, Rosado J, Tran H, Galanis C, Vlachos A, Jedlicka P, Queisser G, Opitz A (2021). Multi-scale modeling toolbox for single neuron and subcellular activity under transcranial magnetic stimulation. Brain Stimul..

[19] Wu T, Fan J, Lee K S, Li X (2016). Cortical neuron activation induced by electromagnetic stimulation: a quantitative analysis via modelling and simulation. J. Comput. Neurosci..

[20] Weise K, Worbs T, Kalloch B, Souza V H, Jaquier A T, Van Geit W, Thielscher A, Knösche T R (2023). Directional sensitivity of cortical neurons towards TMS-induced electric fields. Imaging Neurosci..

[21] Meng Q, Nguyen H, Vrana A, Baldwin S, Li C Q, Giles A, Wang J, Yang Y, Lu H (2022). A high-density theta burst paradigm enhances the aftereffects of transcranial magnetic stimulation: evidence from focal stimulation of rat motor cortex. Brain Stimul..

[22] Peterchev A V, Murphy D L, Lisanby S H (2011). Repetitive transcranial magnetic stimulator with controllable pulse parameters. J. Neural Eng..

[23] Huang Y Z, Edwards M J, Rounis E, Bhatia K P, Rothwell J C (2005). Theta burst stimulation of the human motor cortex. Neuron.

[24] Tysseling V M, Janes L, Imhoff R, Quinlan K A, Lookabaugh B, Ramalingam S, Heckman C J, Tresch M C (2013). Design and evaluation of a chronic EMG multichannel detection system for long-term recordings of hindlimb muscles in behaving mice. J. Electromyogr. Kinesiol..

[25] Cermak S, Meng Q, Peng K, Baldwin S, Mejías-Aponte C A, Yang Y, Lu H (2020). Focal transcranial magnetic stimulation in awake rats: enhanced glucose uptake in deep cortical layers. J. Neurosci. Methods.

[26] Meng Q, Jing L, Badjo J P, Du X, Hong E, Yang Y, Lu H, Choa F-S (2018). A novel transcranial magnetic stimulator for focal stimulation of rodent brain. Brain Stimul..

[27] Paxinos G, Franklin K (2012). Paxinos and Franklin’s the Mouse Brain in Stereotaxic Coordinates.

[28] Seong H Y, Cho J Y, Choi B S, Min J K, Kim Y H, Roh S W, Kim J H, Jeon S R (2014). Analysis on bilateral hindlimb mapping in motor cortex of the rat by an intracortical microstimulation method. J. Korean Med. Sci..

[29] Alekseichuk I, Mantell K, Shirinpour S, Opitz A (2019). Comparative modeling of transcranial magnetic and electric stimulation in mouse, monkey, and human. NeuroImage.

[30] Beining M, Mongiat L A, Schwarzacher S W, Cuntz H, Jedlicka P (2017). T2N as a new tool for robust electrophysiological modeling demonstrated for mature and adult-born dentate granule cells. Elife.

[31] Cuntz H, Bird A D, Mittag M, Beining M, Schneider M, Mediavilla L, Hoffmann F Z, Deller T, Jedlicka P (2021). A general principle of dendritic constancy: a neuron’s size- and shape-invariant excitability. Neuron.

[32] Hodgkin A L, Huxley A F (1952). A quantitative description of membrane current and its application to conduction and excitation in nerve. J. Physiol..

[33] Gomez-Tames J, Hirata A, Tamura M, Muragaki Y (2019). Corticomotoneuronal model for intraoperative neurophysiological monitoring during direct brain stimulation. Int. J. Neural Syst..

[34] Turner R (1986). A target field approach to optimal coil design. J. Phys. D: Appl. Phys..

[35] Roth Y, Zibman S, Pell G S, Zangen A, Tendler A (2023). Revisiting the rotational field TMS method for neurostimulation. J. Clin. Med..

[36] Sinisalo H (2024). Modulating brain networks in space and time: multi-locus transcranial magnetic stimulation. Clin. Neurophysiol..

[37] Jung N H, Gleich B, Gattinger N, Hoess C, Haug C, Siebner H R, Mall V (2016). Quadri-pulse theta burst stimulation using ultra-high frequency bursts—A new protocol to induce changes in cortico-spinal excitability in human motor cortex. PLoS One.

[38] Du X, Choa F S, Summerfelt A, Tagamets M A, Rowland L M, Kochunov P, Shepard P, Hong L E (2015). Neural summation in human motor cortex by subthreshold transcranial magnetic stimulations. Exp. Brain Res..

[39] Labruna L, Merrick C, Peterchev A V, Inglis B, Ivry R B, Sheltraw D (2023). Kilohertz transcranial magnetic perturbation (kTMP): a new non-invasive method to modulate cortical excitability. bioRxiv Preprint.

[40] Sorkhabi M M, Benjaber M, Wendt K, West T O, Rogers D J, Denison T (2021). Programmable transcranial magnetic stimulation: a modulation approach for the generation of controllable magnetic stimuli. IEEE Trans. Biomed. Eng..

[41] Hamada M, Ugawa Y (2010). Quadripulse stimulation–a new patterned rTMS. Restor. Neurol. Neurosci..

[42] Nakatani-Enomoto S (2011). Some evidence supporting the safety of quadripulse stimulation (QPS). Brain Stimul..

[43] Hamada M, Terao Y, Hanajima R, Shirota Y, Nakatani-Enomoto S, Furubayashi T, Matsumoto H, Ugawa Y (2008). Bidirectional long-term motor cortical plasticity and metaplasticity induced by quadripulse transcranial magnetic stimulation. J. Physiol..

[44] Chen R, Gerloff C, Classen J, Wassermann E M, Hallett M, Cohen L G (1997). Safety of different inter-train intervals for repetitive transcranial magnetic stimulation and recommendations for safe ranges of stimulation parameters. Electroencephalogr. Clin. Neurophysiol..

[45] Pascual-Leone A (1993). Safety of rapid-rate transcranial magnetic stimulation in normal volunteers. Electroencephalogr. Clin. Neurophysiol..

[46] Rossi S (2021). Safety and recommendations for TMS use in healthy subjects and patient populations, with updates on training, ethical and regulatory issues: expert guidelines. Clin. Neurophysiol..

[47] Douglas R J, Martin K A (2004). Neuronal circuits of the neocortex. Annu. Rev. Neurosci..

[48] Douglas R J, Martin K A (2009). Inhibition in cortical circuits. Curr. Biol..

[49] Harris K D, Shepherd G M G (2015). The neocortical circuit: themes and variations. Nat. Neurosci..

[50] Yang W, Carrasquillo Y, Hooks B M, Nerbonne J M, Burkhalter A (2013). Distinct balance of excitation and inhibition in an interareal feedforward and feedback circuit of mouse visual cortex. J. Neurosci..

[51] Cruikshank S J, Lewis T J, Connors B W (2007). Synaptic basis for intense thalamocortical activation of feedforward inhibitory cells in neocortex. Nat. Neurosci..

